# The Biological Relevance of Papaverine in Cancer Cells

**DOI:** 10.3390/cells11213385

**Published:** 2022-10-26

**Authors:** Daniella Anthea Gomes, Anna Margaretha Joubert, Michelle Helen Visagie

**Affiliations:** 1 Department of Physiology, School of Medicine, Faculty of Health Sciences, University of Pretoria, Private Bag X323, Gezina, Pretoria 0031, South Africa; 2 Laboratory for Biological Characterisation of Advanced Materials (LBCAM), TTMI, School of Medicine, Trinity College Dublin, D08 W9R Dublin, Ireland

**Keywords:** papaverine, vascular endothelial growth factor, phosphatidylinositol-3-kinase, phosphodiesterase 10A

## Abstract

Papaverine (PPV), a benzylisoquinoline alkaloid, extracted from the *Papaverine somniferum* plant, is currently in clinical use as a vasodilator. Research has shown that PPV inhibits phosphodiesterase 10A (PDE10A,) resulting in the accumulation of cyclic adenosine 3′, 5′-monophosphate (cAMP) that affects multiple downstream pathways, including phosphatidylinositol-3-kinase/protein kinase B (PI3K/Akt), a mammalian target of rapamycin (mTOR) and vascular endothelial growth factor (VEGF). The accumulation of cAMP can further affect mitochondrial metabolism through the activation of protein kinase A (PKA), which activates the mitochondrial complex I. Literature has shown that PPV exerts anti-proliferative affects in several tumorigenic cell lines including adenocarcinoma alveolar cancer (A549) and human hepatoma (HepG-2) cell lines. Cell cycle investigations have shown varying results with the effects dependent on concentration and cell type with data suggesting an increase in cells occupying the sub-G_1_ phase, which is indicative of cell death. These results suggest that PPV may be a beneficial compound to explore for the use in anticancer studies. More insight into the effects of the compound on cellular and molecular mechanisms is needed. Understanding the effects PPV may exert on tumorigenic cells may better researchers’ understanding of phytomedicines and the effects of PPV and PPV-derived compounds in cancer.

## 1. Introduction

Cancer is a non-communicable disease that is characterized by unregulated cell proliferation, evasion of the immune system, cell death induction, continuous angiogenesis and the potential to undergo metastasis [1,2]. Cancer is one of the leading causes of death globally with 10 million deaths accompanied with approximately 19.3 million new cases [1]. Incidence rates for cancer are approximately 19% higher in males than in females worldwide [1]. Consequently, there has been increased interest in cancer research, as well as phytomedicinal research with the intention of developing more effective anti-cancer treatments with fewer side effects [3,4,5].

## 2. Papaverine

**Figure 1:**
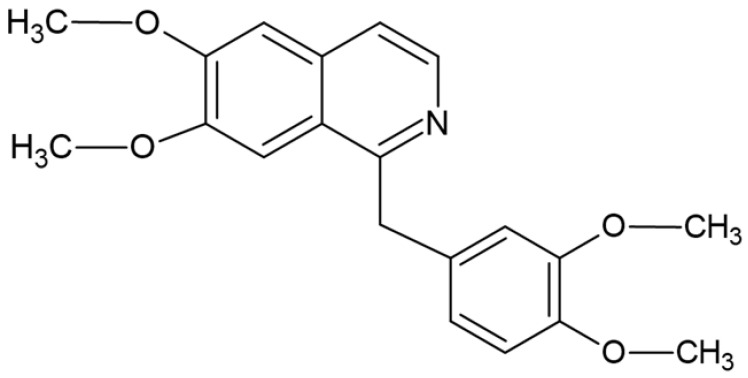
Chemical structure of PPV (Image designed by DA Gomes using ChemSpider (released in 2008, Royal Society of Chemistry, Raleigh, NC, USA).

Papaverine (PPV) is a non-analgesic benzylisoquinoline alkaloid extracted from the *Papaverine somniferum* plant, commonly known as the opium poppy seed plant (poppies) and was first isolated in 1848 (Figure 1) [6]. PPV is found in approximately 0.5%–3% of all the alkaloid content that is possibly obtained from the opium poppy seed, depending on the subtype and continent the poppy seed is harvested from (Table 1) [7,8]. These findings were contradicted by reports that suggest PPV consists of 0.3%–29.7% of all the alkaloid content from the poppy seed [8]. Most of the alkaloids extracted from the opium poppy seed exert analgesic effects including morphine and codeine, however, PPV differs from the opium group of alkaloids both chemically and pharmacologically [8,9]. Whilst most of the main alkaloid compounds isolated from the opium poppy are narcotic and exert an analgesic effect, PPV’s pharmacological use is predominantly as a non-narcotic, non-analgesic smooth muscle relaxant and vasodilator [9,10]. Globally, there are several different areas in which poppies grow, resulting in various different cultivars, climate types and chemical types of the same species [8]. The consequence of this is that the chemical composition of each cultivar can be significantly different and therefore the alkaloid extracts isolated from the plant material can vary [8].

PPV was approved by the Food and Drug Administration (FDA) of the United States as a vasodilator to be predominantly used in the treatment of cerebral vasospasms and coronary circulation [6,11,12,13,14]. However, the mechanisms of action of PPV and the affected pathways is still not yet fully understood [7,15].

The bioavailability of PPV upon oral administration is approximately 30% and the pharmacokinetic half-life in humans is between 1.5 h and 2.5 h [16,17,18,19,20]. The effects exerted by PPV as a vasodilator and muscular relaxant appear to be dose-dependent, with different optimum doses depending on the target tissues including doses of 4 mg/mL, 8 mg/mL and 12 mg/mL used in coronary blood flow investigations that demonstrated increased coronary blood flow and doses of 100 mg/100 mL to 300 mg/100 mL used in patients with cerebral ischemia [12,14,18,21,22,23]. The efficacy of PPV, referring to the extent to which PPV works under optimum circumstances, was found to be approximately 50% with the median lethal dose (LD50) in rats found to be 750 mg/kg [21,22,24]. Possible side effects that have been noted include priapism at doses of 60 mg/mL or higher occurring in approximately 33% of patients [21,22,24]. Approximately 57% of patients risk the occurrence of developing penile fibrosis when administered PPV [21,22,24].

Currently little literature exists regarding the mechanisms of action exerted by PPV in tumorigenic cell lines. The majority of available studies demonstrates the effects induced by PPV on cell viability, cell cycle and cell death resulting in the mechanism of action utilized by PPV in tumorigenic cells remaining elusive [6,7,15,20,25,26,27]. Morphological changes as a result of treatment with PPV have been investigated in prostate cancer with results indicating that lymph node carcinoma of the prostate (LNCaP) cells exhibit morphological changes whilst prostate cancer (PC-3) and androgen-independent prostatic carcinoma (DU145) cells exhibit little to no morphological alterations [25]. In addition, two other known phosphodiesterase (PDE) inhibitors (theophylline and 3-isobutyl-1-methylxanthine) were used to compare morphological alterations in LNCaP-, PC-3- and DU145 cells with the results indicating that exposure to PPV exhibited more prominent morphological changes in LNCaP cells than the other PDE inhibitors [25]. Furthermore, the removal of PPV 6 days post-exposure resulted in little or no changes to the morphological changes observed during exposure, suggesting that the morphological changes are irreversible [25]. Proliferation studies on LNCaP cells indicated that PPV significantly inhibited the proliferation of LNCaP over a period of 6 days when compared to untreated LNCaP cells [25]. Additional studies exploring the effects of PPV on morphology included research conducted on DU145 cells, a triple-negative breast cancer cell line (MDA-MB-231) and an adenocarcinoma alveolar cancer cell line (A549) [20]. Results indicated that after exposure to PPV for 48 h and 72 h, an increase in uncharacteristic morphology and cell debris was observed in a time- and dose-dependent manner in MDA-MB-231, DU145 and A549 cells [20]. Furthermore, MDA-MB-231 cells exhibited a greater increase in uncharacteristic morphology when exposed to PPV than the DU145- and A549 cells [20]. These studies therefore indicate that exposure to PPV can affect morphology in a time- and dose- dependent manner with the extent of the effects observed being cell line specific [20,25].

The effects of PPV on cell proliferation have been investigated in several cell lines, with results indicating the extent of cell proliferation reduction dependent on cell line (Table 2). Studies comparing the maximal reduction in cell proliferation of tumorigenic cell lines to non-tumorigenic cell lines exposed to PPV revealed that non-tumorigenic cells were less affected by PPV [7]. Furthermore, the results varied between the cell lines supporting previous research that suggests that the effects of PPV is cell line-dependent [7]. These findings were further supported on human hepatoma (HepG-2) cells after exposure to PPV for 48 h, where results indicated that the percentage of viable cells decreased to 38% corresponding with an increase in the concentration of PPV [28]. Additional studies investigating the effect of PPV on cell proliferation revealed that PPV reduces cell proliferation in MDA-MB-231, A549 and DU145 cell lines after 48 h to 56%, 53% and 64%, respectively.

Additionally, the effects of PPV cytotoxicity on a human prostate cancer cell line (PC-3) and non-tumorigenic human fibroblast (NHF) cells was investigated after exposure to PPV for 24 h. Results indicated that PPV reduced the cell viability of PC-3 cells by 90% whilst cell viability in the non-tumorigenic NHF cell line was reduced by 2% [29]. This further supports that PPV exerts its effects on tumorigenic cells whilst the effects on non-tumorigenic cell lines are negligible and supports the findings that the effects of PPV is cell line specific [7,29]. In addition to cell line selectivity and dose-dependent effects, results have also indicated that the effects exerted by PPV are time-dependent.

Studies investigating cell cycle and cell death have yielded contradicting results with studies indicating that time and cell line dependence affects cell cycle progression [15,20,29]. The cell cycle is a complex process which involves the growth and proliferation of cells, organ development, regulation of deoxyribonucleic acid (DNA) repair and the possible development of tissue hyperplasia (increase in the amount of organ tissue as a result of proliferation) in response to injury or diseases including cancer [30,31,32]. There are multiple regulatory measures that guide cells through the signaling cascade which ultimately leads to mitosis and thus the formation of two daughter cells [32]. Interphase is one of two subphases of the cell cycle and consists of three subdivisions, namely the gap 1 (G_1_) phase, the synthesis (S) phase and the gap 2 (G_2_) phase [31]. Cells that are in the G_0_ phase perform their differentiated function; they are not actively dividing, but have the potential to undergo cell division [31]. Furthermore, unlike non-tumorigenic cells, tumorigenic cells will continually enter G_0_ and re-enter the cell cycle. Novel compounds are continuously being designed and synthesized to selectively induce cell cycle arrest, whereby tumorigenic cells are unable to enter a subsequent cell cycle phase resulting in induction of cell death [30,31]. Compounds that affect or damage DNA trigger checkpoints that can lead to cell cycle arrest in either G_1_, S or G_2_ phase [31]. In addition, endoreduplication (also known as endoreplication) is a process by which cells that possess DNA damage continue to enter the cell cycle to replicate without dividing, resulting in daughter cells which are polyploids, referring to cells which contain more than two sets of chromosomes [33,34]. This results in cells that can avoid programmed cell death, bypassing the checkpoints.

Reports have indicated that PPV exposure results in aberrant cell cycle abnormalities, with some studies suggesting the accumulation of cells occupying the sub-G_1_ phase whilst other studies indicate G_0_/G_1_ cell cycle arrest or S phase blocks that are dose- and cell line-specific and possibly time-dependent (Table 3) [15,29]. Treatment with PPV for 48 h decreased the percentage of cells occupying the G_0_/G_1_ phase in MCF-7 cancer stem cells and exposure to PPV for 48 h in MDA-MB-231 cancer stem cells increased the percentage of cells in the G_0_/G_1_ phase [15]. Furthermore, previous research conducted on prostate carcinoma (PC-3) cells indicated that 48 h exposure to PPV induced an increase of cells in the sub-G_1_ cell phase, whilst a low percentage of untreated cells occupied the sub-G_1_ phase, indicating an increase of cell death when exposed to PPV [29]. Cell cycle and cell death investigations using propidium iodide (PI) staining and ethanol fixation on MDA-MB-231 cells after exposure to PPV for 72 h exhibited an increase of cells occupying the sub-G_1_ phase and an increase in cells undergoing endoreduplication compared to cells propagated in growth medium. Furthermore, a 72 h exposure to PPV in A549 cells exhibited an increase of cells occupying the sub-G_1_ phase accompanied by an increase in cells undergoing endoreduplication when compared to cells propagated in growth medium, whilst, in DU145 cells, exposure to PPV for 72 h exhibited an increase of cells occupying the sub-G_1_ phase accompanied by an increase in cells undergoing endoreduplication when compared to cells propagated in growth medium [20]. These results indicate that the effects exerted by PPV on cell cycle are dose-dependent and cell line-specific.

Furthermore, some studies have indicated that PPV induces early- and late apoptosis. Data obtained by evaluating the membrane asymmetry using translocation of phosphatidylserine on the cell surface (Annexing V-FITC assay) indicated that exposure to PPV for 48 h induced early and late apoptosis in PC-3 cells in a dose-dependent manner in comparison to untreated PC-3 cells [29]. These results suggest that PPV induces apoptosis rather than necrosis; however, more studies need to be conducted in order to elucidate the effects exerted by PPV in more cell lines to conclude that apoptosis is induced [29]. Furthermore, results following exposure to PPV for 24 h indicated that PPV induces apoptosis in HT-29- and T47D- cells, with little apoptosis induction in HT1080- and NIH-3 T3 cell lines [7]. Thus, it is suggested that apoptosis induction by PPV is cell line-specific and dose-dependent [7,29].

Another effect of the clinical importance that PPV presents is its potential vasodilation effects due to its ability to inhibit phosphodiesterase 10A (PDE 10A), which also accounts for the muscle relaxant effects PPV exerts [14,23,35,36,37]. This is supported by studies that suggested that the mechanism of action of PPV could be the inhibition of phosphodiesterase activity in vascular and uterine smooth muscles [37]. It was further suggested that this effect may cause the build-up of cyclic adenosine 3′, 5′- monophosphate (cAMP) intracellularly [37]. The influence of cAMP on tumor progression, the mitochondria and the transcription of oncogenes, including p53, is extensive with the accumulation of cAMP resulting in increased downstream pathway activity including the phosphatidylinositol-3-kinase/ protein kinase B (PI3K/Akt) pathway [19,38,39,40,41,42,43,44,45,46]. Furthermore, studies suggest that increased PDE 10A correlates to increased pulmonary hypertensive vasculature, suggesting the inhibition of PDE 10A may possibly cause vasodilation. However, the extent of this correlation remains elusive [47]. Research conducted comparing the behaviour of mice treated with PPV with mice treated with deficits in PDE 10A indicated that there was an insignificant difference in mice behaviour and further supports the suggestion that PPV inhibits PDE 10A [19]. However, the effects of PPV on neurotrophic pathways and behavior still need further investigation [48].

## 3. Possible Pathways of Mechanisms of Action

### 3.1. Phosphodiesterases and cAMP

Phosphodiesterases (PDEs) are a family of 11 enzymes which is expressed depending on the environment and generally functions as regulators of cAMP and cyclic guanosine 3′, 5′-monophosphate (cGMP) levels [19,38,39]. In addition, cAMP accumulation potentially may affect the activation of several downstream pathways that can influence tumor progression, the mitochondria and the transcription of several oncogenes including p53 [19,38,39,40,41,42,43,44,45,46].

PDE 10A is expressed in specific tissues including the putamen and caudate nucleus of the brain and the testis, with reduced levels of expression observed in the thyroid and pituitary gland as well as the thalamus and cerebellum [19,38]. PDE 10A has a higher affinity for cAMP than cGMP in comparison to most other members of the PDE family. Furthermore, PDE 10A is the only PDE where the cAMP ligand binds to the GAF domain, which may account for its higher affinity for cAMP [19,38,39,40,43,44]. GAF is an acronym derived from the first three protein families which were identified in the domain, these are the mammalian cGMP-dependent phosphodiesterases, *Anabaena* adenylyl cyclases, and *Escherichia coli* FhlA [49].

**Figure 2:**
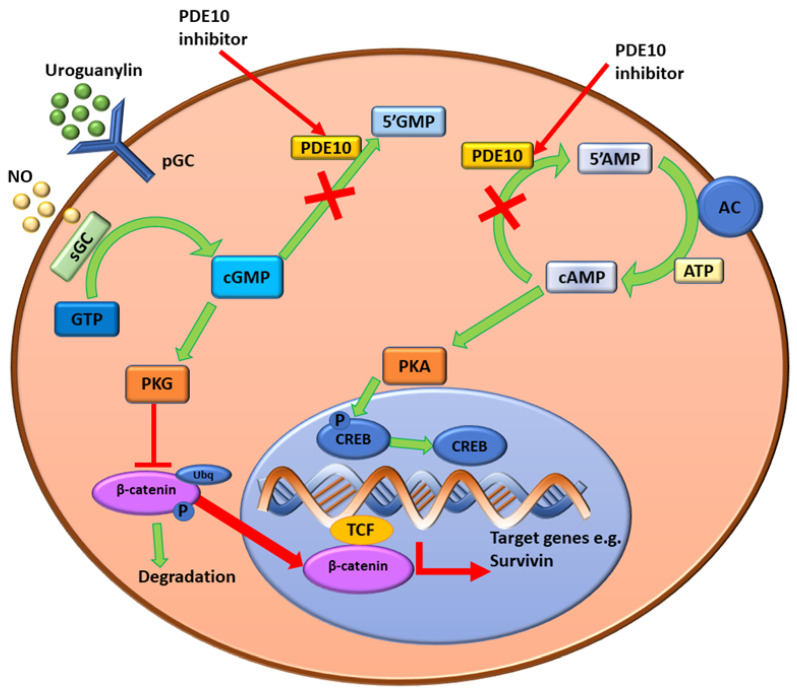
Schematic diagram for the mechanisms underlying the anti-tumor effects of PDE10 inhibition in colon tumor cells. Intracellular cGMP levels are increased due to the inhibition of PDE10A preventing the breakdown to 5′GMP. This activates PKG, which results in the suppression of the expression of β-catenin and inhibits T-cell factor (TCF) transcription of target genes (e.g., survivin). The inhibition of PDE10A prevents breakdown of cAMP to 5′AMP, AC enzymes continue to form cAMP using ATP causing cAMP levels to increase [46]. cAMP activates PKA which phosphorylates CREB. CREB can then activate downstream signaling pathways that either stimulate or inhibit oncogene expression [42]. Image created DA Gomes using Microsoft^®^ office PowerPoint (Microsoft office enterprise 2007, 2006 Microsoft Corporation, Redmond, Washington, DC, USA).

In addition, there is potential for cAMP to antagonize cGMP hydrolysis at lower concentrations of cAMP (half maximal inhibitory concentration (IC_50_) of 0.39 µM) whereas inhibition of cAMP with cGMP requires much higher concentrations (IC_50_ of 14 µM) [38]. The influence of cAMP and cGMP on tumorigenesis is complex, with both molecules and their downstream effects involved in inhibition or stimulation of oncogene expression depending on cell or tissue type [19,38,39,40,41,42,43,44,45,46]. The synthesis of cGMP from guanosine triphosphate (GTP) occurs by the guanylyl cyclase (GC) family of enzymes (Figure 2) [41]. The heme-containing soluble GC (sGC) is found only in the cytoplasm and is activated by nitric oxide (NO) [41]. Subsequently, cGMP activates cGMP-dependent serine/threonine protein kinase (PKG) which thereafter affects several downstream cell signaling pathways including the regulation of β-catenin [41]. The phosphorylation of β-catenin by PKG leads to the degradation of β-catenin or the suppression of β-catenin expression in the nucleus that prevents the T-cell factor (TCF) transcription of target genes such as survivin [41].

The generation of cAMP from adenosine triphosphate (ATP) occurs by the action of the adenylate cyclase (AC) family of enzymes [44,46]. Subsequently, cAMP activates protein kinase A (PKA), a phosphorylating enzyme that cleaves the phosphate groups from transcription factors including the cAMP response element binding protein (CREB) and regulates other cell signaling pathways, including the activation of p27^Kip1^, a cyclin-dependent kinase (CDK) inhibitor (Figure 2) [46]. The main functions of cAMP and cGMP are as secondary messengers, which regulate various functions dependent on tissue type, including the promotion of cell survival pathways [38,41,45,50]. The concentrations of cAMP and cGMP within cells are highly regulated by extracellular signaling which either stimulates their synthesis by AC enzymes or their degradation by PDEs [38,50]. The implication of this is that phosphodiesterase 10A (PDE10A) may selectively regulate cyclic nucleotide levels and therefore regulates cAMP by degrading the cyclic nucleotides to control the levels of cAMP [38,50]. Alterations to these highly regulated signaling pathways can upregulate or downregulate several pathways such as the PKA pathway, which then affects proliferation and cell survival dependent on tissue type, and therefore has implications on tumor progression and tumorigenesis [41]. As a result, PDEs and the cAMP and cGMP cascades have been explored as potential targets for cancer treatments with varying levels of success [41,42,45,50].

The inhibitory effects of PPV on PDE10A have led to several studies that explored the potential use of PPV in in vitro and in vivo in cancer models. Literature indicates that PPV increases cAMP quantities in rat mast cells three-fold and alters the mitochondrial respiration [15,51]. Furthermore, research investigating the effects of PPV on cell morphology and proliferation in prostate cancer cells suggested that elevated cAMP levels in prostate cancer culminates in cell growth arrest [25,52]. These results indicated that exposure to PPV for 15 min before extraction of intracellular cAMP leads to an increase in cAMP levels in prostate cancer (LNCaP) cells when compared to untreated cells [25]. Additionally, morphological alterations, including cells exhibiting smaller cell bodies with long processes or extensions, were increased in LNCaP cells treated with PPV compared to untreated cells and proliferation of LNCaP cells was significantly inhibited, supporting the suggestion that PPV increases cAMP levels and suppresses cell proliferation in prostate cancer [25]. The implication that PPV alters morphology was further supported in MDA-MB-231, A549 and DU145 cells, where exposure to PPV for 48 h and 72 h resulted in aberrant morphology, the extent of which was dependent on cell line [20]. Research has also suggested that the combination of PPV and postaglandin E2 (PGE2) induced morphological alterations and decreased cell proliferation in LNCaP cells by disrupting the cAMP cycle and suppressing the expression of several oncogenes including cellular myelocytomatosis (c-myc) and B cell lymphoma (Bcl) 2, two vital oncogenes in the regulation and control of cell death and apoptotic pathways [15,53,54]. These studies suggest that the inhibitory effects of PPV on PDE10A causes accumulation of cAMP which may affect cell proliferation, morphology and cell survival through several downstream affected pathways.

### 3.2. cAMP in the Mitochondria—Mitochondrial Respiration

**Figure 3:**
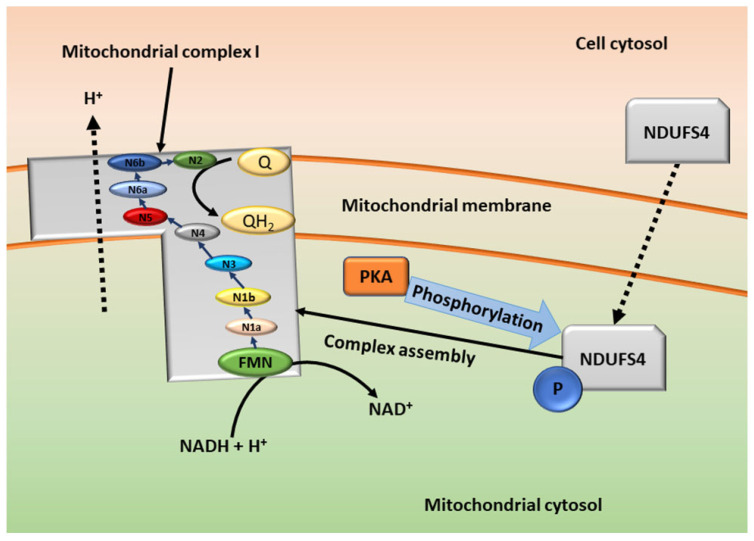
Schematic diagram of the formation and function of the mitochondrial complex I. NDUFS4 is synthesized in the nucleus and transported to the cell cytosol. In the cytosol, PKA can phosphorylate it before it is transported into the mitochondria (not shown) or NDUFS4 is transported into the mitochondrial cytosol where it is phosphorylated by PKA. NDUFS4 then assists in the assembly of the mitochondrial complex I. The mitochondrial complex I can then oxidize NADH to 2NAD, transferring electrons via an electron chain to Ubiquinone (Q). Flavin mononucleotide (FMN) is the entry point for electrons from NADH; electrons are then transferred to iron–sulfur clusters with several different enzymes involved. The pathway of electron transport is indicated by the blue arrows. Ubiquinone is the final electron acceptor and is then reduced by coenzyme Q (coQ) to ubiquinol (QH_2_) [55,56]. Image created by DA Gomes using Microsoft^®^ office PowerPoint (Microsoft office enterprise 2007, 2006 Microsoft Corporation, Redmond, Washington, DC, USA).

The cAMP cascade is involved in the regulation of the entry point of the electron transport chain, the mitochondrial complex I [50]. Mitochondrial complex I consists of several subunits including flavin mononucleotide (FMN) and Ubiquinone (Q), that can be divided into core and accessory subunits, coded by nuclear and mitochondrial deoxyribonucleic acid (DNA) [50]. The effects of core and accessory subunit phosphorylation can be cAMP-dependent and are dependent on the subunit phosphorylated [50,55]. An accessory subunit of complex I, encoded by nuclear DNA, nicotinamide adenine dinucleotide hydrogen (NADH) dehydrogenase [ubiquinone] iron-sulfur protein 4 (NDUFS4) has been investigated to be an area of high mutations in humans that is often associated with neurological disorders [50,56]. Studies have suggested that NDUFS4 is phosphorylated by PKA in the mitochondria resulting in NDUFS4 entering a higher state of energy, or it is phosphorylated in the cytosol before undergoing transmembrane transportation into the mitochondria (Figure 3) [50]. PKA therefore plays an influential role in the activation of the mitochondrial complex I [50,55,56]. Furthermore, studies have shown that PKA activation is influenced by cAMP and therefore PPV may indirectly affect the mitochondrial complex [50,55,56]. In addition to the electron transport chain, the mitochondrial complex I is one of the main sources of reactive oxygen species (ROS) [56]. The increased production of ROS results in oxidative stress, potentially influencing cell survival and proliferation [50,56]. Research investigating the effects of PPV on the production of hydrogen peroxide (H_2_O_2_), as an indicator of oxidative stress in MDA-MB-231, A549 and DU145 cells, showed that PPV increases H_2_O_2_ production after 48 h and 72 h in comparison to cells propagated in growth media [20]. This indicated that ROS production is affected by PPV and results in oxidative stress, which may potentially be a result of the inhibition of PDE10A and cAMP accumulation [20]. This supports previous findings that PDE10A inhibition by PPV results in the accumulation of cAMP, which activates PKA, leading to the phosphorylation of NDUFS4 and ultimately the upregulation of the mitochondrial complex 1 and thus the upregulation of ROS [20,50,56].

Sources of mitochondrial complex I inhibition include over 60 different families of compounds that are categorized into three different classes, Class I/A, Class II/B, and Class C, with the binding site of these inhibitors sharing the same hydrophobic pocket. These inhibitors are grouped in each class according to their kinetic effects on mitochondrial complex I [56]. Rotenone, a well known Class II mitochondrial complex I inhibitor that is used for inhibition of the mitochondrial electron transport, has been used in some studies to compare the effects of PPV on mitochondrial complex 1 in order to establish the mechanism of action of PPV [56,57,58]. Research has suggested that PPV dose-dependently competitively inhibits the activity of mitochondrial complex 1 by blocking rotenone inhibition. Furthermore, literature suggests that PPV is a more potent mitochondrial inhibitor when compared to the activity exerted by rotenone [58]. Results showed that exposure to PPV for 150 min reduced the oxygen consumption rate (changes in oxygen concentration and intracellular pH) to 50% whilst exposure to rotenone (1 µM) for 150 min reduced the oxygen consumption rate to 50% in a murine mammary cancer cell line (EO771) [6,59]. These results indicate that PPV and rotenone act competitively and furthermore suggest that PPV may bind to the rotenone site or the rotenone site is blocked when PPV binds [6]. Whilst this study indicated a possible binding site of PPV, it is still not fully explored, and more research is required on the binding site of PPV. Furthermore, this research showed that PPV can directly inhibit or uncouple the mitochondrial respiratory chain since a panel of 28 tumorigenic and non-tumorigenic cell lines exposed to low concentrations of PPV demonstrated a decrease in mitochondrial function in a short amount of time [6]. The binding site of rotenone has been suggested to be found on the O_2_ side of the NADH dehydrogenase in mitochondrial complex I with many known mitochondrial complex inhibitors binding to the same site, including piericidin A and capsaicin [57]. As it has been shown that the rotenone site is blocked when PPV binds, it is possible that PPV binds to the O_2_ side of the NADH dehydrogenase in mitochondrial complex I [6]. Additionally, researchers suggested that the effects exerted by PPV on the mitochondria are reversible in vitro, allowing the restoration of the mitochondria [6]. In addition, results showed that cells exposed to PPV returned to the baseline OCR, whilst rotenone-treated cells showed no restoration of mitochondrial function [6]. However, further investigations using the phosphodiesterase inhibitor sildenafil citrate on epithelioid carcinoma isolated from the pancreatic duct (PANC-1) cells resulted in increased cAMP levels and did not affect OCR or the mitochondria [6]. Since PPV affects the mitochondrial complex I independently from PDE10A inhibition, it is suggested that PPV has more than one target and affects tumor mitochondrial metabolism [6].

Furthermore, effects of PPV on mitochondrial respiration were explored by means of exposing cells cultured in glucose-free growth media or glucose-containing media in order to establish if cells are dependent on optimal mitochondrial activity to survive glucose deprivation [26]. Results indicated that exposure to PPV exhibited dose-dependent effects on cell viability in colon cancer (DLD1), ER-positive breast cancer (MCF-7) and human glioblastoma (U87) cell lines. Furthermore, the reduction in cell viability was more prominent in PPV-treated cells cultured in glucose-free media in comparison to PPV-treated cells cultured in glucose-containing media [26]. Additionally, the effect of PPV on cell death was investigated using flow cytometry and annexin V, which demonstrated the induction of cell death more prominently in tumorigenic cells exposed to PPV propagated in glucose-free medium when compared to tumorigenic cells exposed to PPV in glucose-containing complete growth medium [26]. This suggested that PPV selectively induces cell death more prominently in glucose-deprived conditions.

### 3.3. mTOR and PI3K

Tumorigenesis is a complex stepwise process with the involvement of various pathways including cAMP, PI3K/Akt and vascular endothelial growth factor (VEGF) pathways playing a significant role in progression. Downstream effects of the PDE10A and cAMP pathways include the PI3K/Akt and extracellular signal-related kinase (ERK) pathways which may then further affect VEGF signaling [19,38,39,40,41,42,43,44,45,46]. There is growing evidence of cross-signaling between the cAMP pathway and the mammalian target of rapamycin (mTOR) pathway and the ERK pathway [60]. The inactivation of several tumor suppressor genes including phosphatase and tensin homolog (PTEN), p53 and necrosis factor 1 (NF1) have been implicated in regulatory associated protein of TOR (raptor)–mTOR activation also referred to as the mTOR complex 1 (mTORC1), suggesting that the increase in cell growth is a result of the raptor–mTOR complex [61].

Studies have suggested that cAMP can either inhibit or stimulate the formation of mTOR complexes depending on cell type [60]. Furthermore, the cAMP and mTOR pathways potentially upregulate several tumorigenic hallmarks including cell cycle progression, cell mobility, cell survival and metastasis [41,42,45,50,60,62]. Consequently, the antiproliferative effects of PPV may be mediated through their effects on cAMP which may further mediate the inhibition of raptor–mTOR signaling through PKA disruptions; however, this mechanism is not fully understood [29,60]. Furthermore, upregulation of mTOR and raptor–mTOR leads to the upregulation of hypoxia-inducible factor 1α (HIF1α) which has been shown to upregulate VEGF ligands and their associated receptors [63,64].

There are five VEGF ligands, including VEGF B, which are associated with hypoxia-dependent angiogenesis and stimulating the survival of vascular cells. Subsequently, tumor progression correlates with increased hypoxia that stimulates VEGF upregulation [65]. Upregulation of VEGF B leads to neovasculature that is not fully developed and therefore is hyperpermeable, and this upregulation of VEGF B been shown to significantly impact vasculature sprouting and the promotion of vasculature survival, allowing the neovasculature developed withing the tumor to survive [65,66,67,68]. However, contradicting results from various literature sources indicate that there are still several questions regarding VEGF activity, since some studies indicate that it functions as a pro-survival molecule and others indicate that it is an angiogenic factor [67,68,69,70,71,72,73,74,75]. The interaction of VEGF B with VEGF R1 is one of several possible modes of action exerted by VEGF B, with research indicating that VEGF B has a higher affinity to VEGF R1 compared to other VEGF ligand receptors [67,68,69,71]. When VEGF B binds to VEGF R1, cell signaling is facilitated, depending on cell type, with several studies indicating that VEGF R1 affects homeostasis and vascular development [67,68,69,71]. Furthermore, overexpression of VEGF R1 has been shown to inhibit VEGF R2 expression and the phosphorylation of extracellular signal-related kinase (ERK) [69]. Research has shown that exposure to PPV for 48 h resulted in the downregulation of VEGF B in MDA-MB231, A549 and DU145 cell lines [62]. Furthermore, the results indicated that exposure to PPV for 48 h resulted in the upregulation of VEGF R1 in MDA-MB-231 and DU145 cell lines [62]. These effects may be mediated by the PDE10A, cAMP, mTOR and PI3K/Akt pathways. However, further investigation into these mechanisms must be conducted [62].

Furthermore, research indicated that exposure to PPV for 48 h results in the upregulation of VEGF R1 in MDA-MB-231 and DU145 cells, whilst VEGF R1 expression was downregulated in A549 cells [62]. This supports findings that demonstrate that VEGF R1 expression is cell type-specific and further suggests that PPV effects on VEGF R1 expression is cell line-specific. Additionally, no observable changes to the expression of VEGF R2 was seen when exposed to PPV for 48 h in MDA-MB-231, A549 and DU145 cells [62]. This indicates that the overexpression of VEGF R1 corelates with decreased VEGF R2 expression [62,69]. Furthermore, it has been suggested that VEGF R2 mediates growth and survival signaling via the PI3K/Akt pathway, which subsequently activates downstream proteins including focal adhesion kinase (FAK) [62,69]. Research has suggested that PPV downregulates the PI3K/Akt pathway [29]. With literature also indicating an association between VEGF R2 expression and FAK signaling, it is possible to suggest that as both VEGF R2 and FAK expression are not affected by PPV after 48 h, the downregulation of the PI3K/Akt pathway occurs by means of alternative signal transduction [62].

**Figure 4:**
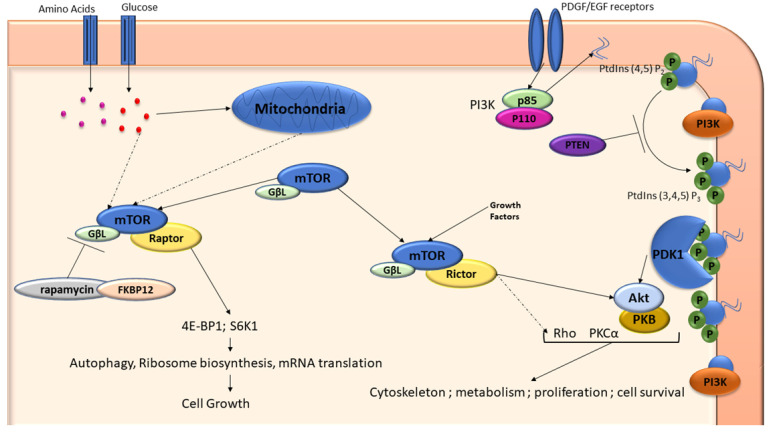
A schematic diagram of the mTOR and PI3K/Akt pathway. Raptor and rictor are two mTOR-interacting proteins that define 2 separate branches of the mTOR pathway. Raptor–mTOR pathway regulates cell growth through interactions with S6K1 and 4E-BP1 proteins. Nutrients can influence the response of the raptor–mTOR branch [61]. The rictor–mTOR pathway regulates Akt/PKB to control cell survival, proliferation, metabolism, and cytoskeleton formation. The binding of growth factors to receptors such as PDGF activates PI3K to generate Phosphatidylinositol (PtdIns) (3,4,5) P_3_ from PtdIns (4,5) P_2_. This recruits the PDK1 kinase and Akt/PKB to the membrane. PTEN is a tumor suppressor gene that regulates the activity of PI3K. Akt/PKB can then be activated by phosphorylation at 2 separate sites [61]. Rictor–mTOR complex phosphorylates Ser473 on Akt/PKB; this may lead to the phosphorylation of Akt/PKB on Thr308. The regulation of this complex is relatively unknown. Solid arrows indicate direct interactions and dash arrows indicate interactions that are indirect [61]. Image created by DA Gomes using Microsoft^®^ office PowerPoint (Microsoft office enterprise 2007, 2006 Microsoft Corporation, Redmond, Washington, DC, USA).

Research exploring the effects of PPV on the mTOR pathway activity was conducted in order to gain further molecular understanding regarding the cellular responses to PPV exposure under conditions of glucose starvation [26]. The function of TOR remains poorly understood, however, literature indicates that TOR is a central component in several signaling networks that regulate cell growth, proliferation and animal size (Figure 4) [61,76]. The complex is composed of mTOR, the G protein β-subunit-like protein (GβL) and regulatory associated protein of TOR (raptor) proteins. The phosphorylation of several regulator proteins, including s6 kinase (S6K1) and eIF-4E binding protein 1 (4E-BP1), is controlled by mTOR, which link the raptor–mTOR complex to the control of mRNA translation [61,76]. A measure of the activity of the raptor–mTOR complex is the phosphorylation state of S6K1 since it is activated by the raptor–mTOR complex through phosphorylation (Figure 4) [61]. In the event of cancer, the inactivation of several tumor suppressor genes including phosphatase and tensin homolog (PTEN), p53 and necrosis factor 1 (NF1) has been implicated in raptor–mTOR activation, suggesting the increase in cell growth is a result of the raptor–mTOR complex [61].

The effects of PPV on the mTOR pathway was investigated using colorectal adenocarcinoma (DLD1), breast carcinoma (MCF-7) and a glioblastoma (U87) cell line. Cells were exposed to PPV in glucose-free growth medium or glucose-containing medium and the phosphorylated-4EBP1, phosphorylated-P70S6K and phosphorylated-S6RP expression was evaluated, which is indicative of mTOR pathway activity [26]. The results suggested that exposure to PPV in glucose-free media resulted in a reduction in phosphorylation of these proteins, further suggesting the inhibition of the mTOR pathway.

Rapamycin-insensitive companion of mammalian target of rapamycin (Rictor) is a hydrophobic motif kinase for protein kinase B (PKB), also known as Akt (Akt/PKB) and plays a significant role in the activation of Akt/PKB (Figure 4) [61]. The rictor–mTOR complex (also referred to as the mTOR complex 2 (mTORC2)) does not bind to FK506 binding protein 12 (FKBP12) as the raptor–mTOR complex does, and its role in the activation of Akt/PKB is a pivotal component of the insulin/phosphoinositide 3-kinases (PI3K) pathway [60]. PI3K is a crucial pathway that regulates cell survival and proliferation [61]. In tumorigenic cells, the Akt/PKB pathway is frequently upregulated due to the loss of the PTEN tumor suppressor gene, resulting in the rictor–mTOR complex being a treatment target. There is growing evidence that the raptor–mTOR complex and its downstream signals repress or affect the PI3K/Akt signaling pathway through phosphorylation, although further investigation into this is required [61]. Research has suggested that PPV induces the phosphorylation of Akt that is dependent on cell line (DLD1, MCF-7 and U87 cell lines) and the glucose levels/availability in media [26]. This further supports the suggestion that PPV affects the mTOR pathway under glucose starvation and further suggests that the action of PPV on tumorigenic cell lines is cell line specific, suggesting that downstream pathways such as the P3K pathway may be affected.

PI3K is a lipid kinase that phosphorylates the 30-hydroxyl group of phosphoinositides, resulting in the formation of the second messenger phosphatidylinositol- 3,4,5-trisphosphate (PIP3), which is critical in the recruitment of Akt for the activation of growth, proliferation and survival signaling [77]. PTEN negatively regulates PIP3 by dephosphorylation, thus, loss of PTEN or other disruptions to the PI3K pathway often result in tumorigenesis [77]. After exposure to PPV for 48 h, results suggested that PI3K and phosphorylated Akt expression was dose-dependently reduced. It was therefore suggested that the effects of PPV may be mediated through the inactivation of NF-kB [29]. Research investigating the effects of PPV on nuclear factor kappa-light-chain-enhancer of activated B cells (NF-kB), as well as PI3K and Akt expression, in PC-3 cells revealed that NF-kB expression was downregulated when exposed to PPV in comparison to the untreated control. It was therefore suggested that the anti-cancer effects of PPV observed were as a result of NF-kB downregulation [29]. NF-kB is an important transcription factor that affects several downstream genes including tumor necrosis factor (TNFα) and platelet derived growth factor (PDGF) [64]. In addition to TNFα and PDGF, NF-kB has been shown to affect PI3K expression. Literature has shown that exposure to PPV for 48 h in PC-3 cells results in the dose-dependent downregulation of PI3K and phosphorylated Akt [29].

### 3.4. HMGB1 and RAGE

High mobility group box 1 (HMGB1) is expressed in many tumor types including gastric adenocarcinomas and its over expression has become a known hallmark of cancer [78]. Research has indicated that the PI3K/Akt pathway is regulated by HMGB1, indicating its significance in tumorigenesis [79]. HMGB1 is a non-histone chromosomal protein involved in DNA replication, transcription, and DNA repair and is involved in cell survival, migration, differentiation and promotion of inflammation [78]. The release of HMGB1 in response to infections or injuries can be passive or active [63]. Passive secretion of HMGB1 into the extracellular space has been found to occur during cell necrosis, as well as active secretion of HMGB1 from inflammatory cells [78]. After secretion of HMGB1, HMGB1 binds to receptors on the cell surface which then typically induces an intracellular response. It has been suggested that the receptor for glycation end products (RAGE) is a significant receptor for HMGB1, which influences adhesion, migration, autophagy and immune responses, mediated through mitogen-activated protein kinase (MAPK), NF-kB and mTOR [63]. This interaction leads to signaling that involves extracellular signal-related kinase (ERK) 1 phosphorylation which ultimately aids in cell proliferation [78]. An increase in HMGB1 in the tumor microenvironment leads to an increase in HMGB1–RAGE interactions. This interaction initiates the phosphorylation of ERK1 which upregulates cell proliferation and can therefore cause cancer regrowth and resistance to therapies [78]. The interaction of HMGB1 with T cell immunoglobulin mucin–3 (TIM-3), an immunoregulatory protein, induces the secretion of VEGF, which promotes tumor angiogenesis. Furthermore, the intracellular cytoplasmic tail molecule of TIM-3 is involved in the recognition of phosphatidylserine (PtdSer) on the surface of apoptotic cells, which signals to immune cells including natural killer cells and mast cells [63].

Tumor research has shown that exposure to PPV in human glioblastoma multiforme (GBM), uppsala 87 malignant glioma (U87MG) and human glioblastoma multiforma tumor (T98G) cell lines inhibits the interaction between HMGB1 and RAGE and suggests that PPV functions as a RAGE suppressor [27,35]. This was done by comparing the cell proliferation of cells exposed to 10 μg/mL HMGB1 to cells exposed to both HMGB1 and PPV. Results indicated that the cell proliferation of cells exposed to HMGB1 only increased by 40% in comparison to the vehicle control, whilst cells exposed to both HMGB1 and PPV decreased cell proliferation by 40% in comparison to the vehicle-treated control cells. Tis indicated that PPV inhibits HMGB1 promoted cell proliferation [35]. In addition, studies have shown that RAGE inhibition induced by PPV may be a beneficial anti-inflammatory compound for the treatment of sepsis with a half maximal effective concentration (EC_50_) of approximately 10.1 µM in monocyte/macrophage-like (RAW264.7) cells [27]. However, the extent of RAGE suppression in vivo was not established and requires further investigation [27].

### 3.5. Vascular Endothelial Growth Factor

There are several hallmarks of tumorigenesis including prolonged proliferation, evading growth suppressors and the induction of angiogenesis [80]. Angiogenesis is the controlled formation of new blood vessels from pre-existing vasculature [65]. During tumorigenesis, the angiogenic switch occurs whereby the tumor grows beyond a sustainable size resulting in the release of growth factors in order to stimulate vessel growth towards regions of hypoxia [65,66,81,82]. Vascular endothelial growth factor (VEGF) is one of the most well-known proangiogenic growth factors. The induction of VEGF expression can be mediated through several growth factors, cytokines and transcription factors including hypoxia-inducible factor 1-alpha (HIF-1α) [67,83]. The effects of VEGF on endothelial cells are reportedly mediated by the PI3K/AKT/mTOR pathways [81,83].

It has been suggested that the effects exerted on the vasculature by PPV is a result of the inhibition of PDE10A, which may aid in inhibiting tumor progression [6]. Additionally, it has been suggested that the cAMP pathway mediates the production of prostaglandin E2 (PGE2), which induces the secretion of VEGF in prostate cancer [84]. Furthermore, investigations exploring the effects of PPV in combination with an antiangiogenic compound, Bevacizumab (also referred to as Avastin), on tumor xerographs in mice with colon cancer (DLD1) cells demonstrated that this combination of compounds was more effective at reducing the rate of tumor growth than either compound did separately, indicating that PPV may exert a greater effect in hypoxic environments [26]. The effects of PPV on tumorigenic cells and VEGF expression are not yet fully understood and more research needs to be conducted.

### 3.6. Apoptotic Pathways

Under stress, cells respond in a plethora of ways including via the expression of molecules that activate cell survival pathways or activate cell death pathways such as apoptosis [85]. The caspase family of cysteine proteases are common death effector molecules of apoptosis [85]. Procaspases are found in the cell cytoplasm or nucleus as inactive proenzymes which can be activated by cleaving various substrates [85]. Caspase activation in the mitochondria is induced by the release of apoptotic factors within the mitochondria including cytochrome *c* and apoptosis-inducing factor (AIF). Activation of cytochrome *c* secretion into the cytosol leads to the activation of caspase 3 through the caspase 9-containing apoptosome complex [85]. As previously discussed, the cAMP cascade can promote proapoptotic pathways dependent on the stressor and cell type, mediated by the phosphorylation of Bcl-associated X protein (BAX) through PKA [50]. BAX is a proapoptotic Bcl related protein that stimulates the expression of cytochrome *c* and leads to the cleaving of caspase 9 and the activation of caspase-dependent apoptosis [50]. It may therefore be possible that the action of PPV on PDE10A and its effects on the cAMP cascade could potentially play a role in the induction of apoptosis [50]. Consequently, the caspase family can be used as markers or indicators of apoptosis.

Research evaluating caspase activity using a homogeneous caspases assay fluorometric kit in breast ductal carcinoma (T47D) cells suggested that PPV does not increase caspase-3 activity [7]. In contrast, studies exploring the effects of PPV on proapoptotic and antiapoptotic proteins using Western blotting indicated that PPV dose-dependently reduces the expression of Bcl-2 proteins and dose-dependently increases Bax expression [29]. Furthermore, PPV dose-dependently increased the release of cytochrome *c* in PC-3 cells suggesting that PPV induces apoptosis through the increase in proapoptotic proteins, including Bax, and the decrease in antiapoptotic proteins, including Bcl-2, as well as the activation of caspases mediated by cytochrome *c* [29]. Additionally, the effects of PPV on the expression go NF-kB and PI3K/Akt were assessed using Western blots. The results suggest that NF-kB expression was significantly downregulated. It was therefore suggested that the effects of PPV treatment were mediated by means of NF-kB inactivation and downregulation of the PI3K/Akt pathway [29]. Despite these studies, the apoptotic pathways induced by PPV are still not fully understood and further research into the apoptotic pathways stimulated by PPV is required.

## 4. Conclusions

In conclusion, papaverine is an active benzylisoquinoline alkaloid component isolated from the poppy seed plant and is FDA-approved and already in clinical use as a vasodilator [9,10]. The interest in PPV in anticancer research is increasing since research on PPV and its effects in tumorigenic cell lines have revealed that exposure to PPV reduces cell viability and induces cell cycle arrests and cell death investigations [6,7,15,20,25,26,27]. However, more research into the mechanism of action of PPV and the signaling pathways affected by PPV is being conducted. To date, it has been suggested that PPV affects PDE10A and the cAMP pathway, PI3K/Akt/mTOR pathway, HMGB1 activity and VEGF expression [6,27,28,29,36,53,62].

It has been suggested that PPV inhibits the expression of PDE10A, which ultimately leads to an upregulation in cAMP and subsequently increases the levels of, and can alter the expression of, several tumor suppressors [15,51]. The effects of PPV on the PI3K/Akt/mTOR pathway are less understood; however, it has been suggested that PPV inhibits the recruitment of Akt to PI3K via PIP3 [26]. Further investigation into this pathway and the effects exerted by PPV on the pathway needs to be explored more as a result. Furthermore, the effects of HMGB1 on the RAGE receptor appear to be disrupted by PPV [27,35]. This results in various intracellular effects including alterations to the PI3K/Akt/mTOR pathway and changes in the expression of several proangiogenic factors, including PDGF and VEGF. However, these effects are still not entirely understood and further investigation on these effects on these pathways needs to be explored.

Consequently, due to PPV possessing antiproliferative effects, through the disruption of cAMP, PI3K/Akt/mTOR and the HMGB1/RAGE pathways, which may lead to the induction of aberrant cell cycle abnormalities possibly culminating in apoptosis, it has been suggested that PPV may be a beneficial compound to explore for use in anti-cancer studies [6,27,28,29,36,53,62]. More insight into the effects of the compound on cell signaling needs to be found. Furthermore, it has been established that the effects of PPV are dose-, cell- and time-dependent [6,7,15,20,25,26,27]. Overall, understanding the effects PPV may exert on tumorigenic cells may better researchers’ understanding of phytomedicines and the effects of PPV and PPV-derived compounds in cancer.

## Figures and Tables

**Table 1:** A breakdown of the main alkaloid contents extracted from *Papaverine somniferum* [8,10].

Alkaloid Name	Alkaloid Content Range within Total Alkaloids (%)	Average Alkaloid Content within Total Alkaloids (%)	Pharmacological Use
Papaverine	0.3–29.7	3.70	Smooth muscle relaxant
Codeine	0.5–25.5	7.69	Used as a cough suppressant and pain killer
Morphine	42.6–87.6	67.9	Dominant alkaloid used as a naturally occurring pain killer
Thebaine	1.9–18.1	5.66	Used industrially to synthesize other pain killers
Noscapine	1.1–41.2	16.05	Cough suppressant

**Table 2:** A summary of the maximal antiproliferative effects exerted by various concentrations of PPV resulting in cell viability in tumorigenic and non-tumorigenic cells.

Cell Line	Highest PPV Concentration Tested (µM)	Cell Viability (%)	Period of Exposure to PPV (h)	Reference
MDA-MB-231	150	56	48	[20]
A549	150	53	48	[20]
DU145	150	64	48	[20]
PC-3	200	15	24	[29]
HT 29	100	35	48	[7]
HT1080	>100	15	48	[7]
T47D	>100	25	48	[7]
LNCaP	100	30	144	[25]
NIH-3 T3	1000	85	48	[7]
NHF	200	98	24	[29]

**Table 3:** A summary of the effects of different concentrations of PPV on the percentage of cells occupying the sub-G_1_ phase in tumorigenic and non-tumorigenic cells.

Cell Line	PPV Concentration (µM)	Cells Occupying the Sub-G_1_ Phase (%)	Period of Exposure to PPV (h)	Reference
PC-3	120	52.4	48	[29]
MDA-MB-231	150	47	72	[20]
A549	150	9.6	72	[20]
DU145	150	24	72	[20]

## Data Availability

No original data is included in this article.
